# Metabolic Engineering of Glycofusion Bispecific Antibodies for α-Dystroglycanopathies

**DOI:** 10.3390/antib13040083

**Published:** 2024-10-07

**Authors:** Xiaotian Zhong, Guoying Grace Yan, Apurva Chaturvedi, Xiuling Li, Yijie Gao, Mahasweta Girgenrath, Chris J. Corcoran, Liz Diblasio-Smith, Edward R. LaVallie, Teresse de Rham, Jing Zhou, Molica Abel, Logan Riegel, Sean K.H. Lim, Laird Bloom, Laura Lin, Aaron M. D’Antona

**Affiliations:** 1 BioMedicine Design, Discovery and Early Development, Pfizer Research and Development, 610 Main Street, Cambridge, MA 02139, USAyijie.gao@roche.com (Y.G.); chris.j.corcoran@pfizer.com (C.J.C.);; 2 Rare Disease Research Unit, Pfizer Research and Development, 610 Main Street, Cambridge, MA 02139, USA

**Keywords:** metabolic engineering, glycofusion bispecific antibody, O-linked matriglycan, dystroglycan, mucin-like domain, dystroglycanopathies, laminin

## Abstract

**Background:** α-dystroglycanopathies are congenital muscular dystrophies in which genetic mutations cause the decrease or absence of a unique and complex O-linked glycan called matriglycan. This hypoglycosylation of O-linked matriglycan on the α-dystroglycan (α-DG) protein subunit abolishes or reduces the protein binding to extracellular ligands such as laminins in skeletal muscles, leading to compromised survival of muscle cells after contraction. **Methods:** Surrogate molecular linkers reconnecting laminin-211 and the dystroglycan β-subunit through bispecific antibodies can be engineered to improve muscle function in the α-dystroglycanopathies. This study reports the metabolic engineering of a novel glycofusion bispecific (GBi) antibody that fuses the mucin-like domain of the α-DG to the light chain of an anti-β-DG subunit antibody. **Results:** Transient HEK production with the co-transfection of LARGE1, the glycoenzyme responsible for the matriglycan modification, produced the GBi antibody only with a light matriglycan modification and a weak laminin-211 binding activity. However, when a sugar feed mixture of uridine, galactose, and manganese ion (Mn^2+^) was added to the culture medium, the GBi antibody produced exhibited a dramatically enhanced matriglycan modification and a much stronger laminin-binding activity. **Conclusions:** Further investigation has revealed that Mn^2+^ in the sugar feeds played a critical role in increasing the matriglycan modification of the GBi antibody, key for the function of the resulting bispecific antibody.

## 1. Introduction

The sarcolemma surrounding a skeletal muscle fiber is closely associated with an extracellular basement membrane layer [1,2]. The basement membrane contains an internal lamina and an external reticular lamina composed of many secretory proteins, such as members of the laminin family and the collagens. The basal lamina is linked directly to the cell membrane through transmembrane receptors such as dystroglycan (DG) [3], which provides a mechanical scaffold for muscle fiber adherence. Genetic alternations of some of these proteins can lead to defects such as early embryonic lethality and congenital muscular dystrophy. α-dystroglycanopathies are collectively referred to as a subgroup of rare congenital muscular dystrophies with muscular dystrophy as well as central nervous system and ocular abnormalities [4,5].

The DG protein, a heterodimeric protein composed of a highly glycosylated extracellular alpha subunit (α-DG) and a transmembrane beta subunit (β-DG), is generated through a post-translational cleavage from a common precursor encoded by the *DAG1* [3,6]. Both subunits are the core components of the Dystrophin-associated Glycoprotein Complex, which establishes a transmembrane stress resistance link between the extracellular matrix and intracellular dystrophin and cytoskeleton during muscle activity [7]. This specific interaction is mediated by matriglycan and its binding protein, a major one being laminin 211, skeletal muscle-specific laminin. The generation process of matriglycan includes a unique and complex O-linked mannosylation glycan, which contains phosphorylation, lipid bridge, and a disaccharide repeat of gluconic acid (GlcA) and xylose (Xyl) [4]. This matriglycan disaccharide modification is synthesized by the like-acetylglucosaminyltransferase proteins LARGE [8,9,10,11]. They are bifunctional glycosyltransferases that have the activities of xylosyltransferase (Xyl-T) and glucuronyltransferase (GlcA-T) and therefore the ability to polymerize Xyl-GlcA repeats. The matriglycan has been referred to as a “functional modification” because it is required for the interaction with extracellular matrix ligands and for the maintenance of sarcolemmal integrity in skeletal muscle.

Since the binding of α-DG to extracellular matrix proteins, such as laminin-211 in the basal lamina of skeletal muscles, is essential for maintaining membrane stability and thereby the survival of sarcolemma during muscle activity [12], the therapeutic strategies for α-dystroglycanopathies have been either replacing the non-functional hypoglycosylated α-DG with purified matriglycan-O-glycosylated α-DG proteins (replacement therapy) or constructing a functional mimic that restores the linkage between the laminins and the sarcolemma. The efficacy of the protein replacement therapeutics in a mouse model has been demonstrated by the local muscle injection of the recombinant α-DG protein, which shows some levels of protection of sarcolemma from laser-induced damage [12]. A bispecific antibody scaffold [13,14] which can bind simultaneously to two different protein targets like laminin-211 and α-DG, has been reported to serve as a surrogate linker between the basal lamina and the sarcolemma [15]. The treatment with this bispecific antibody has demonstrated improved muscle function and protection from exercise-induced damage in the mouse model of α-dystroglycanopathy [15].

In this study, we have generated a novel glycofusion bispecific (GBi) antibody that fuses the mucin-like domain of the α-DG to the N-terminus of the light chain (LC) of an anti-β-DG antibody. This new fusion protein is expected to bind to laminin-211 through the matriglycan on the mucin-like domain of the α-DG fusion while linking the β-DG via the anti- β-DG antibody for achieving the connection between the basal lamina and the sarcolemma. In order to produce the active mucin-like domain of α-DG in the GBi antibody with a proper matriglycan modification, the LARGE gene was co-transfected along with the DNAs encoding the GBi antibody during transient HEK293 production. However, the levels of matriglycan modification of the GBi antibody produced under such a condition were very low, and the GBi antibody exhibited a low binding towards laminin. Unexpectedly, when the GBi antibody was produced under a so-called “glyco-optimized condition” in which there was an addition of a feed mixture of uridine, galactose, and manganese ion (Mn^2+^) to the culture medium, the O-linked matriglycan modification in the GBi antibody was found to be dramatically enhanced. The resulting GBi antibody also displayed a much-improved binding activity toward laminin-211. The protein fractionation experiments further confirmed that the increased binding activity correlated well with the degree of the matriglycan modification. Additional investigations into the contributions of the sugar feed components to the functional production of the α-DG matriglycan modification have revealed a critical role of Mn^2+^ in metabolically engineering the functional GBi antibody.

## 2. Materials and Methods

### 2.1. DNA Constructs

**Figure 1:**
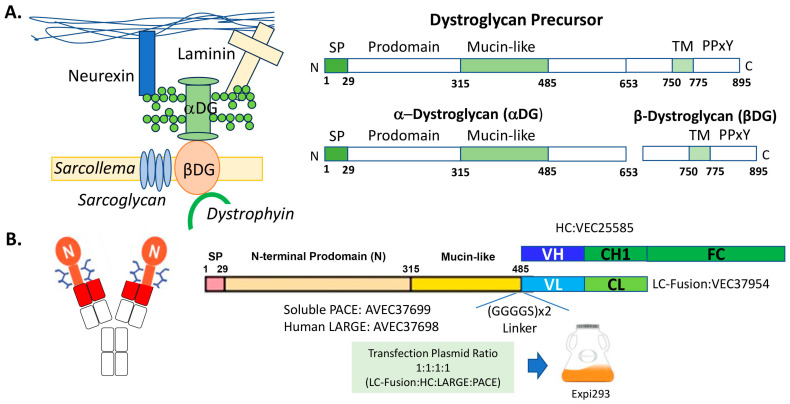
Producing a glycofusion bispecific antibody for muscle dystrophy. (**A**) Schematic drawings of the dystrophin-associated glycoprotein complex and dystroglycan (DG) protein [4,5,18]. (**B**) Schematic drawings of the glycofusion bispecific antibody and the construct designs. The red rectangles in the left schema represent the N-terminal variable domain of an anti-β-DG light chain (VL) or the N-terminal variable domain of the heavy chain (VH). The mucin-like domains are the orange rectangles with the glycan drawings. A linker of (GGGGS)×2 is inserted between the mucin-like domain and the VL. The oval shape with “N” is the N-terminal prodomain of the DAG1 protein. CH1 is antibody heavy chain constant domain 1, and CL is antibody light chain constant domain (CL). Fc contains antibody heavy chain full hinge region and the constant domain 2 and 3. For protein production, the resulting construct DNAs were transfected along with the accessory DNAs encoding human LARGE1 and soluble PACE enzymes into Expi293 cells.

All DNA was gene-synthesized and cloned into a cytomegalovirus (CMV) promoter-based expression vector by Eurofins Genomics Blue Heron (Bothell, WA, USA). Briefly, as shown in Figure 1B, the DNA encoding the heavy chain (HC) of an anti-β-DG antibody was cloned into the CMV vector to form VEC25585. The DNA encoding the mucin-like LC fusion, which is composed of a signal peptide, an N-terminal prodomain, and a mucin-like domain of DAG1 [3] [NP_001159400.3 (aa1-485)] with a (GlyGlyGlyGlySer)×2 [(GGGGS)×2] linker fused to the LC of the anti-β-DG antibody, was cloned into the CMV vector to form VEC37954. The DNA encoding a soluble form of PACE [16] [NP_001276752.1 (aa1-715)] was cloned into the CMV vector to form AVEC37699. The DNA encoding the human LARGE1 [17] (NP_001349878.1) was cloned into the CMV vector to form AVEC37698.

### 2.2. Cell Culture and Transfection

Mammalian Expi293F^TM^ cells were cultured in a Kuhner^TM^ incubator shaker (Adolf Kuhner AG, Basel, Switzerland) with 8% CO_2_ at 37 °C in EXP293^TM^ medium (Thermo Fisher Scientific, Waltham, MA, USA) with a rotation of 120 rpm. A large-scale Expifectamine^TM^-based transient transfection process [19] was used for the GBi antibody production. Briefly, the plasmid DNAs encoding the HC (VEC25585), the mucin-like domain LC fusion (VEC37954), the soluble PACE (AVEC37699), and the human LARGE1 (AVEC37698) in the ratio of 1:1:1:1 at total 1 mg/L were mixed with Expifectamine^TM^ (1:2.7) for a 15 min incubation. The mixtures were inoculated with 3.5 × 10^6^/mL cells with a viability of 99%. 5 mL/L of enhancer-1, 50 mL/L of enhancer-2, as well as supplemental feed UMnG (4 mM uridine, 16 µM MnCl_2_, 40 mM galactose, Sigma-Aldrich, St. Louis, MO, USA) and ManNAc (N-Acetylmannosamine, Sigma-Aldrich, St. Louis, MO, USA, 2 mM) were added 16 h post-transfection. Conditioned media (CM) were harvested after a five-day culture at 37 °C and filtered with 0.2 µm for purification.

### 2.3. Immunoblotting Analysis

The monoclonal antibody IIH6C4 that recognizes carbohydrate epitopes of α-DG [7,20] were purchased from Millipore Corporation (mouse IgM monoclonal antibody, Cat#05-593, 1:400. Billerica, MA, USA). Anti-mouse IgM-HRP was purchased from Southern Biotech (Cat#1021-05, Birmingham, AL, USA). Goat anti-human IgG Fc cross-adsorbed secondary antibody was purchased from Thermo Fisher Scientific (Cat#31125, 1:1000). Donkey anti-goat IgG HRP-conjugated antibody was purchased from Biotechne R&D Systems (Cat#HAF109, Minneapolis, MN, USA). iBind^TM^ Western device (Cat#SLF1000, Thermo Fisher Scientific) and iBind^TM^ Western Starter Kit (Cat#SLF1000S, Thermo Fisher Scientific) were utilized for immunoblotting according to the manufacturer’s protocols. SuperSignal^TM^ West Pico PLUS Chemiluminescent Substrate was purchased from Thermo Fisher Scientific (Cat#34579). The immunoblotting images were captured by Azure c600 Imager (Azure Biosystems, Dublin, CA, USA).

### 2.4. Protein Purification

The 0.2 µm-filtered CM was captured using MAbSelectSure columns (Cytiva, Marlborough, MA, USA), and the flowthrough was reloaded on MAbSelectSure LX columns (Cytiva) for additional capture, as described previously [21]. The protein was analyzed using both analytical scales, YMC-Pack, Diol 200, and Diol 300 (Shimadzu Corporation, Kyoto, Japan). The protein materials were further polished using a preparative scale of 160 mL YMC-pack Diol 300. The load was 4.1 mL/5 mg of the neutralized proA eluate with a running buffer of 20 mM NaPO_4_, 400 mM NaCl, pH 7.2. Multiple fractions were analyzed using the analytical scale YMC-pack Diol-300 and intact/reduced 4–12% SDS-PAGE analysis. Final samples were passed through Natrix^®^ Q Recon Mini devices (MilliporeSigma, Burlington, MA, USA) with an effective membrane volume of 0.2 mL for reducing endotoxins.

### 2.5. Enzyme-Linked Immunosorbent Assays (ELISA)

Plates were coated with 100 ng/well of recombinant β-DG in 8.1 mM Na_2_HPO_4_, 1.47 mM KH_2_PO_4_, 150 mM NaCl, 2.7 mM KCl pH 7.2 (PBS) with 1 mM Ca^2+^/1 mM Mg^2+^ (Ca/Mg) at 4 °C overnight (Corning costar half area REF 3690), blocked with 180 µL PBS with Ca/Mg 3% BSA at room temperature for 1 h. Then, the plate was washed with PBS with Ca/Mg four times. 300 nM GBi antibody or control antibody (in PBS with Ca/Mg 3% BSA) at 25 µL were added to each well and incubated at room temperature for 1 h. The plate was washed with PBS with Ca/Mg four times. Mouse laminin 211 (Cat# 23017-015, Thermo Fisher Scientific) titrate from 10 nM (in PBS with Ca/Mg 3% BSA) at 25 µL was added to each well and incubated at room temperature for 1 h. The plate was washed with PBS with Ca/Mg four times. Rabbit anti-laminin antibody (Cat#L9393, MilliporeSigma, Burlington, MA, USA) at 1:140 (in PBS with Ca/Mg 3% BSA), 25 µL was added to each well and incubated at room temperature for 1 h. The plate was washed with PBS with Ca/Mg four times. The Donkey anti-goat IgG HRP-conjugated antibody (Biotechne R&D Systems, Minneapolis, MN, USA) at 1:5000 (in PBS with Ca/Mg 3% BSA) was added. The plate was washed with PBS with Ca/Mg four times. The TMB substrate was added. The reaction was stopped with 0.18 M sulphuric acid.

### 2.6. Liquid Chromatography–Mass Spectrometry (LC-MS)

As described previously [19,21], for intact and reduced GBi antibody LC-MS analyses, protein samples were deglycosylated with PNGase F for 2 h at 37 °C for intact analysis. For reduced analysis, after deglycosylation, protein samples were reduced with 50 mM DTT (Thermo Fisher Scientific) in the presence of 5 M guanidine HCl (Thermo Fisher Scientific) for 1 h at 37 °C. Prepared protein samples were processed using an ACQUITY UPLC I-Class PLUS System (Waters, Milford, MA, USA) and Waters UNIFI software (version 1.9.4.053). Each sample was injected at 250 ng and separated over a BioResolve mAb Polyphenyl reverse-phase column (450 Å, 2.7 µm) held at 65 °C. Mobile phases A and B were LC-MS Grade 0.1% formic acid in Water and LC-MS Grade 0.1% formic acid in acetonitrile (Thermo Fisher Scientific), respectively. Each run was performed at 0.400 mL/min with the following gradient % B settings: 0 min. 5% B, 3 min. 5% B, 6 min. 85% B, 7 min 85% B, 7.10 min. 95% B, 9 min 95% B, 9.10 min. 5% B, 10 min. 5% B. The ACQUITY RDa Detector for mass spectrometry was set to full scan and positive ion mode with high mass range scanning (400–4000 *m*/*z*). The cone voltage for intact analysis was 70 V and 45 V for reduced analysis. The data were processed using Maximum Entropy settings.

### 2.7. Statistical Analysis

The reported variance is reflective of the observed mean ± standard error of the mean from the two independent experiments. Two-way ANOVA statistical analysis was performed using GraphPad Prism version 9 software (GraphPad Software, San Diego, CA, USA). Two-way ANOVA statistical analysis was performed using GraphPad Prism version 9 software (GraphPad Software, San Diego, CA, USA).

## 3. Results

### 3.1. Producing a GBi Antibody as a Functional Mimic for Restoring the Linkage between the Laminin and the Sarcolemma

As shown in Figure 1A, the *DAG1*-encoded precursor protein contains a signal sequence (SP, 1–29), an N-terminal prodomain (30–314), a mucin-like domain (315–485), a C-terminal extracellular domain (486–749), a transmembrane domain (750–775) and a cytosolic tail (776–895) [3,4]. The 895-amino-acid DG precursor is post-translationally cleaved into noncovalently associated α and β subunits at Ser654. The 43 kDa β-DG subunit consists of a 96-amino-acid N-terminal extracellular region, a single transmembrane domain, and a cytoplasmic domain with a dystrophin-binding site (the PPxY motif) for the cytoskeleton interaction. The β-DG protein unit serves as an axis at the plasma membrane through which the extracellular proteins are tightly linked to the cytoskeleton. The α-DG subunit contains three distinct domains: the N-terminal prodomain required for the matriglycan modification in the mucin-like domain as the binding and anchoring sites for the LARGE1 enzyme [22], the central highly O-mannosylated mucin-like domain, and the C-terminal globular domain involved in the interaction with the β-DG subunit. Figure 1B shows the design of the GBi antibody construct: the N-terminal prodomain and the mucin-like domain of the α-DG were fused to the N-terminus of the LC of an anti-β-DG antibody. The N-terminal prodomain of the α-DG subunit is known to be removed by protein convertases like PACE protease between Arg312 and Gln313 [22,23,24]. Therefore, an accessory vector encoding the soluble PACE protease was co-transfected along with the DNAs encoding the HC and the α-DG mucin-like domain LC fusion during the transient Expi293 production. Since the LARGE1 enzyme is responsible for the matriglycan modification, an accessory vector encoding the LARGE1 gene was also co-transfected. This production process was referred to as the standard procedure for GBi antibody production.

### 3.2. Glyco-Optimized Procedure Produced the GBi Antibody with a Dramatically Enhanced Matriglycan Modification

**Figure 2:**
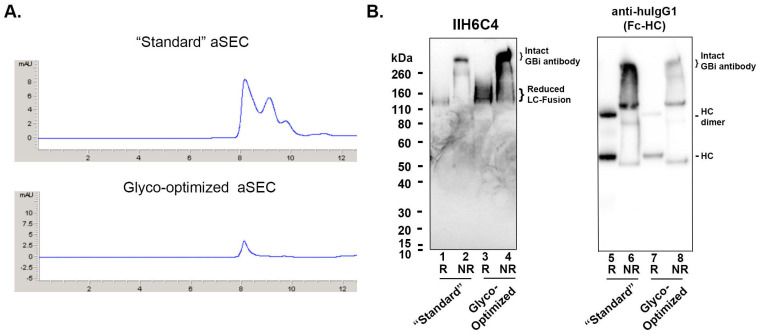
The glyco-optimized procedure produced the GBi antibody with a dramatically enhanced matriglycan modification and a better aSEC profile in HEK293 cells. (**A**) Analytical Diol-200 SEC profiles of GBi antibody derived from the standard production procedure (“standard”) and from the glyco-optimized procedure (Glyco-optimized). (**B**) Immunoblottings of monoclonal antibody IIH6C4 that recognizes the matriglycan epitopes of α-DG and anti-human IgG1 Fc. Equal volumes of the ProA eluates purified from the conditioned media generated by both production procedures (Lane 1, 2, 5, & 6 from the “Standard” procedure; lane 3, 4, 7, & 8 from the “Glyco-Optimized” procedure) were analyzed in the 4–12% SDS-PAGE under the conditions of reduced (R) and non-reduced (NR). The immunoblotting procedure was performed as described in Section 2.

As described in the Materials and Methods, the CM from the transfected Expi293 cell culture under the standard production procedure described above was harvested on Day 5. The CM was purified by the protein-A (ProA) affinity chromatography. The expression titer for the GBi antibody was estimated to be ~12 mg/L. The purified protein was analyzed by an analytical size-exclusion chromatography (aSEC) on Diol-200 resin. As shown in Figure 2A, the aSEC data showed a dominant peak (P1) along with two smaller late migrating peaks, suggesting the existence of low-molecular-mass species. To determine the matriglycan content of the GBi antibody protein, an immunoblotting experiment was performed with monoclonal antibody IIH6C4, which specifically recognizes the matriglycan carbohydrate epitopes of α-DG [7,20]. As shown in Figure 2B lane 1, under reduced conditions, a protein band around 120 kDa was detected. This is consistent with the estimated molecular weight (MW) of the α-DG mucin-like domain LC fusion in which there should be a 74 kDa from the aglycosylated mature protein without the cleavage of the N-terminal prodomain plus additional molecular mass from the O-linked matriglycans and the potential N-glycans at Asn141 [7,20,25]. Since there is no O-linked matriglycan modification in the HC, the 52 kDa HC band was not visible in the immunoblotting of but butIIH6C4 detected in the immunoblotting with anti-human IgG1 Fc (lane 5, an HC dimer band was also visible). Under non-reduced conditions, a dominant band of over 260 kDa was detected in the immunoblotting with IIH6C4 (Lane 2), which was also consistent with the MW of the dimeric form of the GBi antibody with matriglycan modification. This dominant band was also detected in the immunoblotting of anti-human IgG1 Fc (Lane 6).

The GBi antibody is a glycoprotein with both N-glycans and O-glycans in the mucin-like domain LC fusion [3,4]. It has been previously shown that adding sugar feeds and cotransfecting glycogenes can enhance galactosylation and sialylation during transient expression [26,27]. Therefore, a glyco-optimized transfection procedure was performed to increase glycan modification. UMnG (a sugar mixture with uridine, galactose, and Mn^2+^) and ManNAc (a precursor for sialic acids) were added post-transfectionally, whereas pJazz, a plasmid DNA encoding a galactosyltransferase GT1 and a sialyltransferase ST6, was co-transfected along with the target DNAs described in the standard procedure above. Under this glyco-optimized transfection procedure, the expression titer was estimated to be ~1 mg/L, about 10-fold lower than that of the standard procedure. However, as shown in Figure 2A, the aSEC profile of the glyco-optimized procedure-derived materials was much more homogeneous than that of the standard procedure-derived materials, suggesting a better product quality. Unexpectedly, the signals on the immunoblotting with IIH6C4 for the glyco-optimized procedure-derived materials (Lanes 3 and 4) were significantly enhanced in comparison to those from the standard procedure-derived samples (Lane 1 and 2), which should presumably contain 10-fold more loaded protein (Lanes 7 and 8 vs. 5 and 6). These data indicate an increase in matriglycan modification in the GBi antibody derived from the glyco-optimized procedure than those from the standard procedure.

**Figure 3:**
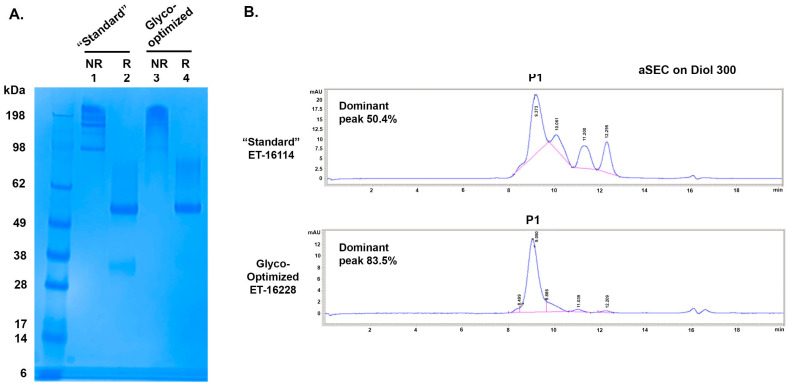
SDS-PAGE and aSEC of GBi antibody derived from either the standard production procedure (“Standard”) or the glyco-optimized production procedure (Glyco-optimized). (**A**) Coomassie-stained SDS-PAGE of purified GBi antibody. Lane 1 & 2 were from the “Standard” procedure and Lane 3 & 4 were from the “Glyco-optimized” procedure. (**B**) aSEC on Diol300 column.

To further characterize these GBi antibody proteins, 2 L of CM each from the standard procedure and the glyco-optimized procedure were purified as described in Section 2. ~23 mg of the GBi antibody protein was purified from the CM of the standard procedure (ET-16114), whereas ~4 mg was purified from the CM of the glyco-optimized procedure (ET-16228). When the samples were analyzed in SDS-PAGE (Figure 3A), the HC matched the expected MW (48.9 kDa unglycosylated form confirmed by the LC-MS) in both batches under the reduced condition. The mucin-like domain LC-fusion (expected unglycosylated MW 73.65 kDa) was smeared up to ~100 kDa with more protein density of the high MW species for the glyco-optimized derived samples (lane 4 vs. 2). Under the non-reduced condition, the protein materials in both batches smeared up to 300 kDa whereas those derived from the standard procedure had more low MW species (lane 1 vs. 3).

When the purified proteins were analyzed by the Diol 300 aSEC (Figure 3B), four peaks were detected for the GBi antibody produced by the standard procedure with 50.4% P1, whereas the majority of the purified materials derived from the glyco-optimized procedure was P1 (83.5%). The GBi antibody derived from the glyco-optimized procedure possessed fewer low-MW species than those derived from the standard procedure. These results together suggest that the additional steps in the glyco-optimized procedure, either the sugar feeds or the glycogenes co-transfection, could result in increased matriglycan modification and better aSEC profiling. The 10-fold lower expression level of the GBi antibody in the glyco-optimized procedure could be due to the modification processes for the mucin-like domain during the production.

### 3.3. The Glyco-Optimized Procedure-Derived GBi Antibody Displayed Dramatically Improved ELISA Activities of the β-DG-Laminin Bridging ELISA and IIH6C4 ELISA

**Figure 4:**
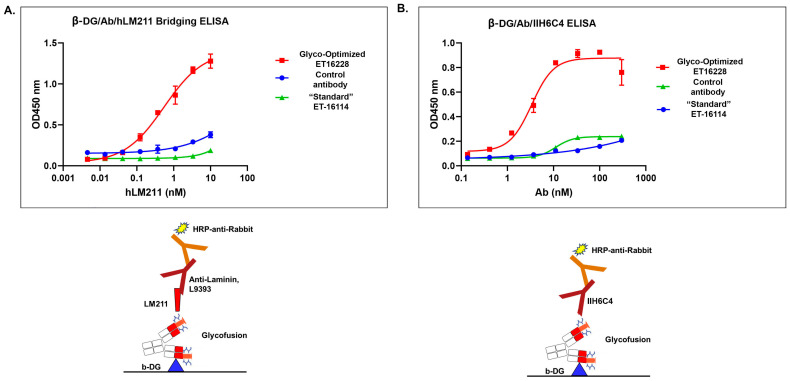
The glyco-optimized procedure-derived GBi antibody displayed dramatically improved β-DG-laminin bridging ELISA and IIH6C4 ELISA activities. The ELISA assays were performed as described in Section 2. Protein samples were immobilized on the plates at equal amounts during ELISA. (**A**) β-DG/Antibody (Ab)/human Lamin-211 (hLM211) bridging ELISA (n = 2 ± S.D.). (**B**) β-DG/Ab/IIH6C4 ELISA (n = 2 ± S.D.).

Matriglycan is a functional modification that is required for the interaction between the extracellular matrix ligands and the α-DG subunit. To characterize the functional binding of the GBi antibody, the purified proteins derived from both batches were assayed in the β-DG-Laminin bridging ELISA. As described in the Section 2, the GBi antibody was immobilized on the plates through the binding to the β-DG protein, and then the laminin-211 was added. The detection of the lamin-211 binding to the GBi antibody was through rabbit anti-Laminin L9393 and HRP-conjugated anti-rabbit antibody. As shown in Figure 4A, the glyco-optimized procedure-derived protein (ET16228) exhibited high binding activity whereas the standard procedure-derived protein (ET16114) had very little binding activity. When the purified proteins were assayed in the β-DG/Ab/IIH6C4 ELISA in which the GBi antibody was immobilized on the plates through the binding to the β-DG protein and antibody IIH6C4 was added, the data in Figure 4B showed that the glyco-optimized procedure-derived GBi antibody displayed the markedly increased IIH6C4 binding activity. This result is consistent with the immunoblotting data of IIH6C4 shown in Figure 2B. Since the monoclonal antibody, IIH6C4 recognizes carbohydrate epitopes of α-DG [7,20], the higher IIH6C4 binding activity of ET16228 over that of ET-16114 was presumably due to the possessing more matriglycan modification.

### 3.4. Further Characterization of the GBi Antibody with a Large-Scale Production under the Glyco-Optimized Procedure

**Figure 5:**
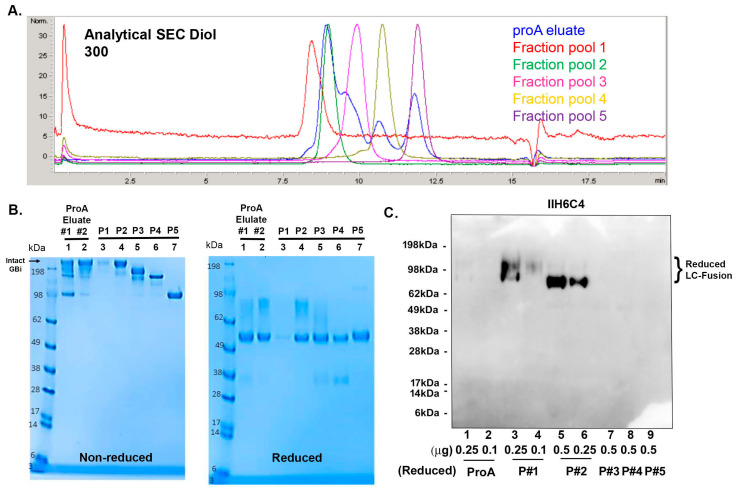
Further characterization of the GBi antibody with a large-scale production under the glyco-optimized procedure. (**A**) Analytical SEC on Diol 300 with ProA eluates, Fraction pool 1 (P#1), Fraction pool 2 (P#2), Fraction pool 3 (P#3), Fraction pool 4 (P#4), and Fraction pool 5 (P#5). (**B**) Coomassie-stained SDS-PAGE of ProA eluates (Lane 1 & 2), Fraction pool 1–5 (Lane 3–7). (**C**) Immunoblotting of IIH6C4 on ProA eluates (Lane 1 & 2) and P#1–5 (Lane 3–9).

**Figure 6:**
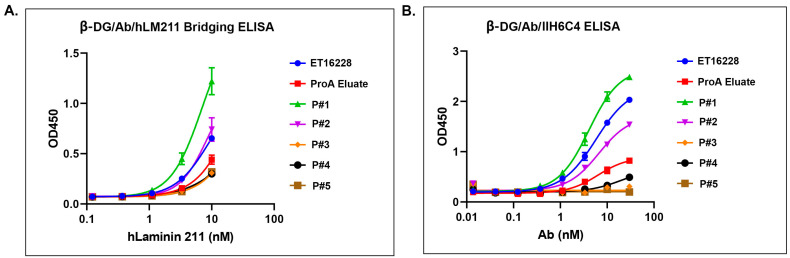
Different pools of GBi antibody displayed a different activity in the β-DG-laminin bridging ELISA and IIH6C4 ELISA. (**A**) β-DG/Ab/hLM211 bridging ELISA (n = 2 ± S.D.). (**B**) β-DG/Ab/IIH6C4 ELISA (n = 2 ± S.D.). Protein samples were immobilized on the plates at equal amounts during ELISA.

To further characterize the GBi antibody derived from the glyco-optimized procedure, 14 L CM was generated, and the protein was captured using two rounds of proA purification. A total of 69 mg of protein was captured from both purification batches. Because the aSEC profile on the Diol 300 column (Figure 5A) showed increased resolution, the protein batches were purified on a preparative Diol 300 column, as described in Section 2. The fraction pools were analyzed by aSEC (Figure 5A), SDS-PAGE (Figure 5B), and immunoblotting with IIH6C4 (Figure 5C). ProA eluates and preparative SEC pool 1–5 all contained the same HC (Figure 5B, reduced, lanes 1–7), but different sizes of the mucin-like domain LC-fusion were detected for pool 1–3 (Lanes 3–5), nearly no LC fusions were detected for pool 4 and 5 (Lanes 6 and 7). These results are consistent with the non-reduced data (Figure 5B, non-reduced lane 1–7), with decreased MWs from pool fractions 1–5. These data are aligned with the result that different pools also contained different signal intensities of the IIH6C4 immunoblotting (Figure 5C, lane 3–6 vs. lane 7–9). These results together indicated that the different retention times of peaks from these pools were due to the different extents of the matriglycan modification in the GBi antibody. As shown in Figure 6, the pools of these purified GBi antibody protein displayed different activities toward Laminin-211 binding and IHH6C4 staining, consistent with the observation that the stronger the IIH6C4 staining signals, the higher the laminin-binding activity.

### 3.5. It Is the Addition of Sugar Feeds That Increases Matriglycan Modification of GBi Antibody

**Figure 7:**
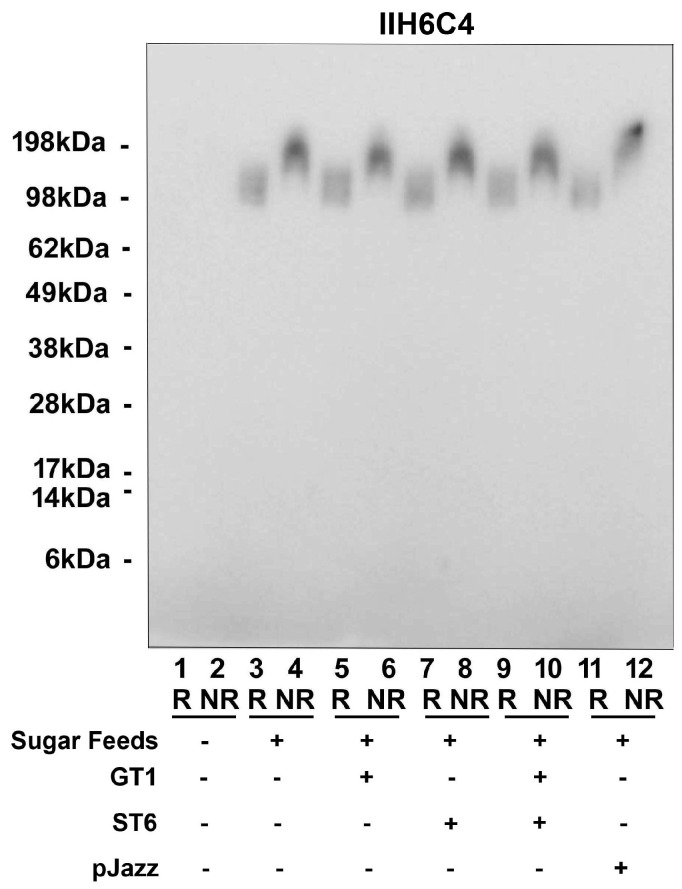
It is the addition of sugar feeds that increases matriglycan modification of GBi antibody. Co-transfecting either GT1 alone, ST6 alone, GT1 and ST6 together, or in a single vector pJazz, along with the GBi antibody construct into Expi293, were performed. Sugar feeds were added post-transfectionally as indicated. Immunoblotting with IIH6C4 was carried out.

Since the glyco-optimized procedure contains additional steps compared to the standard procedure, such as the sugar feeds and different glycogenes, we dissected various production conditions to determine the critical step, such as sugar feeds alone, plus GT1 alone, ST6 alone, or GT1/ST6 combined, or in a single vector pJazz. All conditions tested had a similar expression titer ranging from 1 mg/L to 4 mg/L, as well as cell viability from 44.5% to 62.6%. As shown in Figure 7, as long as the sugar feeds were present, the matriglycan modification was enhanced significantly regardless of the presence or absence of the cotransfection of glycogenes.

### 3.6. Mn^2+^ Played a Critical Role in Producing Functionally Active GBi Antibody

**Figure 8:**
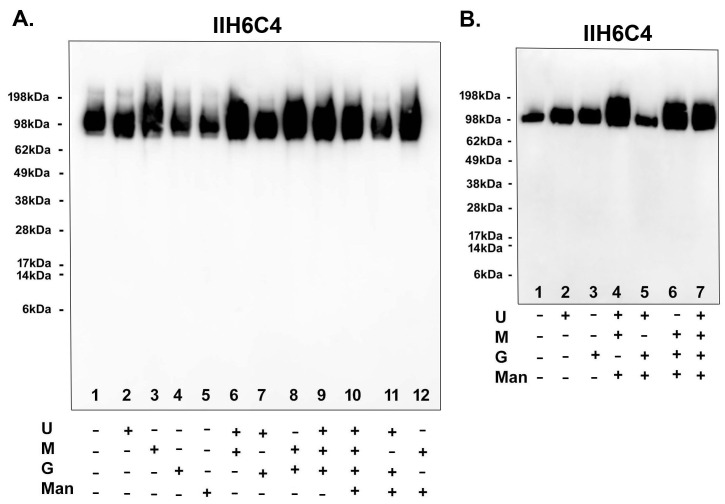
Mn^2+^ played a critical role in producing functionally active GBi antibodies. The GBi antibody constructs were transfected into Expi293 cells. The components of the sugar feeds [uridine (U), manganese ion (M), galactose (G), ManNAc (Man)] were added separately and combined as indicated (**A**,**B**). Immunoblotting with IIH6C4 was performed.

Since adding sugar feeds alone was enough to increase matriglycan modification, we set out to ascertain the most critical factor(s) in the sugar feeds for the modification. As shown in Figure 8A, adding uridine, galactose, or ManNAc alone has no enhancement effect [Lane 1 (no feeds) vs. 2 (uridine only), 4 (galactose only), and 5 (ManNAc only), whereas adding Mn^2+^ alone (lane 3) could enhance the matriglycan modification. Feeds lacking Mn^2+^ [lane 7 (uridine + galactose) and Lane 11 (uridine + galactose + ManNAc)] also had no enhancement effect. Whenever Mn^2+^ was present [lane 6 (uridine + Mn^2+^), 8 (galactose + Mn^2+^), 9 (uridine + galactose + Mn^2+^), and 12 (ManNAc + Mn^2+^)], the enhancement effect was observed. Consistently, in a separate experiment shown in Figure 8B, there was no enhancement when Mn^2+^ was not present in the sugar feeds [lane 5 (uridine + galactose + ManNAc) vs. 1 (no feeds), 4 (lacking galactose), 6 (lacking uridine), and 7 (full feeds)]. These results have together demonstrated that Mn^2+^ indeed played a critical role in producing functionally active GBi antibodies.

## 4. Discussion

This study has attempted to generate a novel engineered GBi antibody to be potentially used as a generalized approach for improving the conditions in the various subtypes of α-dystroglycanopathies with high genetic heterogeneity. Utilizing the laminin-211 binding capability of the matriglycan on the recombinant mucin-like domain of the α-DG fused to an anti- β-DG antibody, the resulting GBi antibody could serve as a surrogate molecular linker that might rescue the loss of binding between the laminin and the α-DG in those subtypes of α-dystroglycanopathies [4,5]. During the production process of the GBi antibody, we found that the functional activity of the α-DG mucin-like domain component required further metabolic engineering. Co-transfecting glycogene LARGE did not enhance the binding activity to laminin-211, but the addition of sugar feeds to the cell culture was critical for producing sufficient matriglycan modification and functional binding. While the glyco-optimized protocol described previously [26,27] can enhance galactosylation and sialylation, the finding in the study indicates that the process also had an impact on O-linked mannosylation, specifically the matriglycan with a disaccharide repeat of GlcA and Xyl. The sugar feeds contain galactose which could potentially be converted into UDP-GlcA [28] utilized by the LARGE enzymes. The protocol described in this study is a new version of the glyco-optimized protocol, which requires the cloning of the LARGE1 gene and soluble PACE gene into a CMV-promoter-vector and their co-transfection along with the GBi antibody. The soluble PACE could remove the N-terminal prodomain of DAG1 after the LARGE1-mediated matriglycan modification. These designs aimed to engineer a functional GBi antibody that can engage the laminin-211 and β-DG. Further investigation has revealed that it is the Mn^2+^ that plays an important role in the metabolic engineering process. While developing a glycofusion bispecific antibody is not a novel problem, the novelty of this work is the requirement for engineering a functional DG mucin-like domain fusion with an uncommon O-linked matriglycan on the resulting GBi antibody. In addition to co-expression of the LARGE1 gene, medium manipulation during mammalian cell culturing was critically required. The biochemical analysis confirmed that the different extents of matriglycan modification correlated with the various binding activity toward laminin-211. This engineering challenge is different from the sialylation issue frequently encountered by other glycofusion proteins, which affects mostly pharmacokinetics and circulatory half-life [29]. To our knowledge, this study is the first such report that metabolic engineering is required for the functionality of a bispecific antibody.

The requirement of Mn^2+^ is likely attributed to the LARGE function. LARGE1 utilized in this study is a type II transmembrane protein localized in the Golgi with two distinct luminal enzymatic domains: a domain-1 (residues 138–413) with a xylosyltransferases activity and a domain-2 (residues 473–742) with a glucuronyltransferase activity [10]. It has been shown that Mn^2+^ has a crucial role in the activity of LARGE1 coordinated by two conserved DXD motifs in domain-1 and one in domain-2 [30,31]. Mutating either one of the two DXD motifs in the first catalytic domain had no effect on the Golgi localization, but both constructs abolished the catalytic activity completely [31]. Mutating the third DXD motif in the second catalytic domain caused mislocalization to the ER, likely affecting protein folding. All these observations indicate that the important role of Mn^2+^ binding motifs for the matriglycan modification. Our finding that the addition of more Mn^2+^ to the cell culture could significantly boost the matriglycan modification has provided direct evidence for the critical role of Mn^2+^ in matriglycan modification. Since Mn^2+^ is present in the normal cell culture medium, the requirement of more Mn^2+^ for matriglycan modification suggests that the concentration of Mn^2+^ in the Golgi lumen might not be sufficient for the full enzymatic activities of the LARGE enzymes. Further investigation, such as manipulating LARGE on Mn^2+^ binding sites and a direct biochemical or kinetic analysis of the enzymatic reaction, should follow in order to understand the mechanistic details.

One potential advantage for the GBi antibody over the bispecific designs targeting laminin and DG is the diverse binding capacity of the matriglycan on the mucin-like domain of the α-DG to multiple extracellular matrix ligands such as laminin, agrin, neurexin, and perlecan [3]. The matriglycan in the mucin-like domain of the α-DG is known to be critical for the binding to laminin-G domain-containing extracellular matrix ligands, and therefore, the interaction between basement membrane and epithelial cells [4,24,32]. The matriglycan is a unique type of O-mannosylation [33] that was first confirmed in the α-DG in mammals [34]. It is initiated by GalNAc-β1,3-GlcNAc-β1,4-Man-Ser/Thr, with phosphorylation at position 6 of the mannose residue. The phosphorylated trisaccharide on the α-DG is linked to the [GlcA β1-3-Xyl-α1,3) disaccharide polymer via a novel phospho-ribitol moieties [24,35]. The negatively charged polysaccharides contribute to the basic patches in the laminin domains [36]. The interactions between dystroglycan and its extracellular binding partners rely on the extent of matriglycan modification [18], therefore the production method based on the finding that the concentrations of Mn^2+^ can modulate matriglycan modification might provide a new way to produce GBi antibody with different degrees of matriglycan modification. This could modulate the binding activity of the α-DG mucin-like domain and might result in a specific interaction with various partners.

It has been noted that the SEC data shown in Figure 5A (from the glyco-optimized procedure) was similar to those shown in Figure 2A (from the standard procedure). This observation is likely due to the scale-up effect from the production of the 2 L to that of the 14 L. Even for purification, the aSEC analysis on the protein derived from the expression titer analysis (Figure 2A) showed differences from those derived from the 2L-scale process (Figure 3B). In the scale-up production during transient expression, the transfection efficiency for the genes encoding the LAGRE enzyme, along with those for target genes, decreased significantly, and their co-expression within individual transfected cells also decreased. The Mn^2+^ distribution in the cell culture could also be substantially affected during the scale-up process. The protein variability during the scale-up transient production is common, which is one of the drawbacks compared to the stable cell production [19,37,38,39]. The reproducibility of the benefit of matriglycan modification with the glyco-optimized method was confirmed and shown in Figure 7 and Figure 8. For fewer variations, stable CHO production is the preferred methodology. Yet this procedure is typically initiated when final lead molecules are identified. For the stage of protein engineering, the rapid production of transient expression provides a significant value to the lead molecule discovery process.

In summary, aberrant O-glycosylation in the α-DG protein can be caused by congenital mutations in at least 17 other genes besides the *DAG1* [24]. Loss of the binding ability to function as a receptor for extracellular matrix ligands for α-DG occurred in these congenital/limb-girdle muscular dystrophies could all be potentially rescued by different formats of bispecific antibodies. The unique design of the GBi antibody reported in this study should enable the physiological binding functions of the α-DG protein for a broader biological consequence. In addition, because the C-terminal domain of α-DG and the extracellular domain of β-DG interact noncovalently, the anti-β-DG bispecific arm of the GBi could provide an additional level of modulation and advantage over the protein replacement therapeutics of the recombinant α-DG protein. Discovering the metabolic engineering requirement for the GBi antibody should greatly facilitate future therapeutics investigation and application in α-dystroglycanopathies. While this study focuses on the biochemical engineering and the production of the GBi antibody, the in vivo functional investigation should be the next step. Systemic delivery and local muscle injection into the LARGE^myd-3J^ mice for muscle damage analysis have been published previously [12,15]. These reports indicate that when the purified matriglycan-glycosylated α-DG was injected systemically, very little change was detected in muscle tissues, presumably due to rapid clearance by the mannose or asialoglycoprotein receptors on the cells in the reticuloendothelial system. However, by the local muscle injection, the recombinant α-DG protein showed protection efficacy of sarcolemma from laser-induced damage [12]. On the other hand, the antibody component of the GBi antibody might improve the circulatory half-life of the mucin-like domain of α-DG through FcRn recycling [40]. All these factors should be considered carefully when the future in vivo investigation is designed.

## Data Availability

Data are contained within the article.
